# Wiring up pre-characterized single-photon emitters by laser lithography

**DOI:** 10.1038/srep31135

**Published:** 2016-08-10

**Authors:** Q. Shi, B. Sontheimer, N. Nikolay, A. W. Schell, J. Fischer, A. Naber, O. Benson, M. Wegener

**Affiliations:** 1 Institute of Applied Physics, Karlsruhe Institute of Technology (KIT), 76128 Karlsruhe, Germany; 2 Nano-Optics, Institute of Physics, Humboldt-Universität zu Berlin, Newtonstraße 15, D-12489 Berlin, Germany; 3 Department of Electronic Science and Engineering, Kyoto University, Kyoto Daigaku-Katsura, Nishikyo-ku, 615-8510 Kyoto, Japan; 4 Institute of Nanotechnology, Karlsruhe Institute of Technology (KIT), 76344 Eggenstein-Leopoldshafen, Germany

## Abstract

Future quantum optical chips will likely be hybrid in nature and include many single-photon emitters, waveguides, filters, as well as single-photon detectors. Here, we introduce a scalable optical localization-selection-lithography procedure for wiring up a large number of single-photon emitters via polymeric photonic wire bonds in three dimensions. First, we localize and characterize nitrogen vacancies in nanodiamonds inside a solid photoresist exhibiting low background fluorescence. Next, without intermediate steps and using the same optical instrument, we perform aligned three-dimensional laser lithography. As a proof of concept, we design, fabricate, and characterize three-dimensional functional waveguide elements on an optical chip. Each element consists of one single-photon emitter centered in a crossed-arc waveguide configuration, allowing for integrated optical excitation and efficient background suppression at the same time.

To bring practical optical quantum information processing to life^1^,^2^, single-photon sources^3^,^4^,^5^,^6^, waveguides and splitters^7^,^8^, filters, as well as single-photon detectors^9^,^10^ need to be integrated into functional quantum-optical chips. The fabrication of such chips, which will likely be hybrid in nature^1^,^2^,^11^, is a demanding task. In free-space optics, using mirrors on an optical table, it is straightforward to couple a given pre-selected single-photon emitter into an optical setup. Doing likewise on an optical chip is a formidable task^1^,^2^. Pioneering experiments^12^ have localized and characterized single-photon emitters based on self-organized semiconductor quantum dots by optical microscopy and spectroscopy. By using alignment markers, the subsequent electron-beam lithography could be aligned with respect to these emitters. More recently, even *in-situ* lithography of basic light collecting structures around quantum dots was reported^13^,^14^. Other experiments have moved nanodiamonds containing nitrogen-vacancy (NV) centers as single-photon emitters to desired locations on surfaces by means of an atomic-force microscope^15^ or with tungsten micromanipulators^16^.

A versatile and simple alternative approach is to use polymer photonic wire bonds to connect different optical components^17^,^18^ – just like cables in electronics. First experiments using randomly distributed quantum emitters have also been performed^19^.

Here, we describe a reliable and scalable approach to first localize and characterize single-photon emitters and then perform aligned optical laser lithography in three dimensions with sub-micrometer precision.

Figure 1 schematically illustrates the localization-selection-lithography procedure. Nanodiamonds containing NV centers are spun onto a 5 μm thick layer of a negative-tone photoresist. Subsequently, the nanodiamonds are covered by a second layer of photoresist.

Two requirements regarding the photoresist are crucial. Fulfilling both of them simultaneously is highly nontrivial. First, the photoresist must be solid as the nanodiamonds could move in a liquid. However, most high-end photoresists for 3D optical laser lithography are liquid^20^. Second, the photoresist must exhibit low background fluorescence – prior to exposure as well as after polymerization.

We meet these requirements by introducing a dedicated solid negative-tone photoresist, which shows substantially less background fluorescence than common photoresists (see Methods and Fig. S1). Moreover, the fluorescence from the polymerized photoresist is six times *lower* than the background fluorescence of the commercial silica glass substrate (LH24.1, Carl Roth) underneath, see Fig. 2.

The setup consists of a continuous-wave laser (561 nm wavelength) that is tightly focused through the glass substrate *via* a 100× microscope oil-immersion lens (numerical aperture NA = 1.4, see Methods) and confocal detection. By raster scanning of the sample with respect to the focus (see Fig. 1a) by means of a 3D piezoelectric translation stage, we acquire two-dimensional PL maps. A typical example is depicted in Fig. 1b. Bright spots in these PL maps are candidates for single-photon emitters. To select from the candidates, the PL is fiber-coupled into a Hanbury Brown and Twiss setup (see Methods). Typically, we use an optical excitation power of 100 μW and acquire data at one location for just one minute. A resulting typical second-order correlation function *g*^(2)^(*τ*) versus time delay *τ* taken at the location indicated by the white circle in Fig. 1b is depicted in Fig. 1c. The fit (orange curve) to these raw data yields photon anti-bunching with *g*^(2)^(0) = 0.14 < 0.5, evidencing single-photon emission. The spatial coordinates of all locations where *g*^(2)^(0) is substantially smaller than 0.5 are recorded. We can characterize about 50 emitters in one hour, of which typically 10 are of the quality shown in Fig. 1c.

Most importantly and quite amazingly, we have not found any indication that the photoresist is exposed by this excitation with tightly focused green laser light.

We note in passing that this pre-selection process is not restricted to NV centers in nanodiamonds. For example, silicon-vacancy centers in nanodiamonds with narrower spectral emission are emerging^21^. Furthermore, one could also select with respect to charge state, quantum efficiency, and dipole orientation^22^. In principle, any other type of emitter is possible.

To write into the photoresist 3D structures which are aligned with respect to the pre-selected single-photon emitters, we focus a frequency-doubled femtosecond fiber laser at 780 nm center wavelength through the same microscope lens (see Fig. 1d and Methods). The foci of the green and the infrared laser are carefully aligned with respect to each other in three dimensions by scanning 100 nm diameter gold beads through the foci and detecting the scattered light. The alignment is checked routinely, but is sufficiently stable over periods of several days. Altogether, this means that the catalogued positions of the 3D piezoelectric translation stage form a *common* three-dimensional coordinate system for the laser pre-characterization and the subsequent laser writing. After laser writing of essentially arbitrary 3D microstructures^23^, the unexposed photoresist is removed in the development process. Electron micrographs of fabricated samples are depicted in Fig. 1e,f. Each of them contains one pre-characterized single-photon emitter right in the waveguide crossing, with a fabrication yield close to 100%.

The motivation for considering the three-dimensional crossed-arc structure is twofold. First, it collects and guides the photons before they are launched into the glass substrate for subsequent far-field detection. Second, it allows for integrated excitation of the emitter and simultaneous background suppression. We now discuss the theoretical design before coming back to the experiments.

Several groups have studied the problem of photon collection from single emitters on or in optical fibers, fiber tapers, or waveguides^24^,^25^,^26^,^27^,^28^. Crucial factors are the emitter-waveguide coupling and guiding losses. A key parameter to tune both is the waveguide radius: If it is too small, only a single mode is guided, but most of the mode energy flows outside of the waveguide, hence the coupling to an emitter inside is weak. If the waveguide radius is too large, the waveguide becomes multi-mode and the collection efficiency is again low. To investigate these aspects in more detail, we have performed finite-element calculations based on the vector Maxwell equations (see Methods).

Figure 3a shows calculated intensity distributions at a fixed emitter vacuum wavelength of 680 nm for a well matched (middle), a smaller (left), and a larger (right) waveguide radius *r* and for a fixed arc radius of *R *= 5 μm. The collection efficiency versus waveguide radius and wavelength is shown in Fig. 3b. In the range of the NV emission spectrum (630 nm to 800 nm), best values are obtained for waveguide radii between 200 nm and 300 nm. Figure 3c shows a cut through these data. The maximum collection efficiency is about 80%. The oscillatory intensity variations are likely due to reflections at the glass interface and the waveguide crossing.

Due to fabrication limitations and the inherently asymmetric focus^20^,^23^, the radius of the fabricated waveguides is *r*_||_ = 0.2 μm parallel to the (*xy*) substrate plane and *r*_⊥_ = 0.4 μm in the normal (*z*) direction, as estimated from electron micrographs.

In almost all experiments so far, waveguide-coupled photon emitters have been excited *via* free-space laser beams. The reason is that excitation light, which is guided in a waveguide as well, would induce too much background fluorescence and Raman scattering, spoiling the single-photon signal. In order to facilitate fully integrated excitation, we have designed the crossed-arc waveguide structure. The excitation light guided in one arc excites a single NV center in a nanodiamond in the middle of the crossing point. The second crossed waveguide efficiently collects the emitted single photons, but only inefficiently collects background accumulated in the excitation waveguide. In order to show this functionality, we use the writing setup in a modified way such that we can optically pump *via* any one of the four waveguide ports and detect at any other port. Wide-field images are shown in Fig. 4a,b. Measured *g*^(2)^(*τ*) traces taken on one example structure are depicted in Fig. 4c,e. The respective excitation and detection geometries are illustrated by the insets. For excitation and detection *via* one port of the orthogonal waveguide in panel c, the fit to the raw data yields *g*^(2)^(0) = 0.21 (0.26 for the other port of the orthogonal waveguide). As expected, when using only a single waveguide in Fig. 4e, more background fluorescence is accumulated (also see Fig. 4b), leading to a deteriorated value of *g*^(2)^(0) = 0.62. Thus, the value of *g*^(2)^(0) = 0.21 for detection at the orthogonal port represents a three-fold improvement. Saturation curves are shown in Fig. 4d,f, exhibiting maximum single-photon emission rates around 40,000 count/s, which can be compared with corresponding typical maximum values of 250,000 counts/s for the setting shown in Fig. 1. From this comparison, we roughly estimate that the collection efficiency is around 30% (see Methods). A comparison of the PL signals for the reference structure *without* a single-photon emitter (blue curves) again shows the improved background suppression due to the crossed-arc waveguide geometry.

In conclusion, we have introduced a localization-selection-lithography approach and a low-background-fluorescence photoresist to fabricate three-dimensional quantum optical functional elements. The method is highly scalable, possesses high yield, and can be fully automated. A next step could be to wire-up several quantum emitters *via* one waveguide to realize an efficient source of photon Fock states with *N* > 1. In this respect, silicon-vacancy centers^29^ in nanodiamonds may be more appropriate than NV centers. The waveguides could also be aligned with respect to the *a priori* randomly oriented emitter dipoles. Another attractive possibility is to introduce a second fabrication step, for example partial metallization in order to fabricate pre-aligned three-dimensional plasmonic antennas^30^ to enhance single-photon emission or microwave antennas to perform optically detected magnetic resonance (ODMR)^31^. Finally, our platform is fully compatible with microfluidics. Therefore, besides quantum optics, our approach could be useful for advanced nano-sensors, in which photon generation, collection, as well as optically-enhanced ODMR would all be integrated in one multi-functional element.

## Methods

Photoresist: The photoresist contained pentaerythritol triacrylate (PETA) as monomer (Sigma-Aldrich) with 3% weight fraction Irgacure 819 as photoinitiator (BASF). Polymethyl methacrylate (PMMA) was used as received (Sigma-Aldrich), dissolved in acetone, and subject to an ultrasonic bath for one hour. PETA was mixed with the PMMA solution using a volume ratio of 7:3. After rapid evaporation of the acetone, this spun-on mixture becomes solid, such that the locations of all embedded nanodiamonds are fixed. After completing the laser writing, the samples were rinsed with acetone and water for development. The nanodiamonds (Microdiamont AG) were used as delivered. They were mixed with Acetone in a volume fraction of 1:10 and ultra-sonicated for 30 minutes. According to the supplier, the nanodiamond ‘size’ nominally ranged between 0–50 nm, the median was 25 nm. The characterization has been performed in the setup described below.

**Figure 1:**
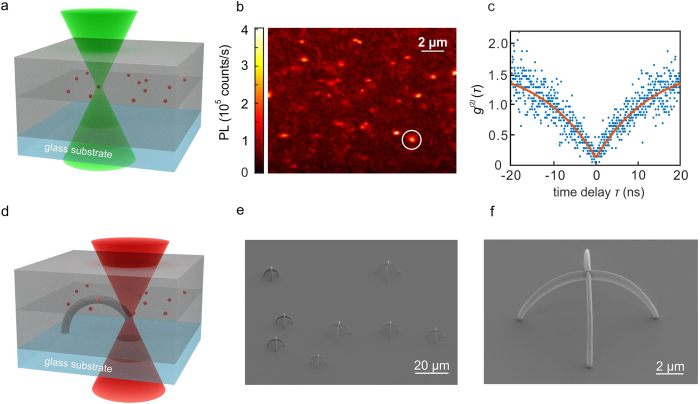
Localization of single-photon emitters and aligned 3D lithography. (**a**) Scheme of the photoresist sandwich. First, a 5 μm thick solid photoresist is spin-coated on a glass substrate. Second, nanodiamonds containing NV centers suspended in isopropanol are spin-coated on top. Third, another thick layer of solid photoresist is added. As a result, the single-photon emitters are located in one plane parallel to the substrate surface within the volume of the photoresist. At this stage, the sample is optically excited by a 561 nm wavelength continuous-wave laser (green), tightly focused to a diffraction-limited spot, and raster scanned. (**b**) Resulting confocal photoluminescence (PL) intensity image. (**c**) Example of a second-order correlation function *g*^(2)^(*τ*) evidencing single-photon emission from the encircled point in (**b**). Excitation power is 100 μW. Similar data are taken at many other locations as well. (**d**) Using femtosecond pulses centered at 780 nm wavelength, 3D microstructures are written, each of which is aligned with respect to one of the various pre-localized and pre-characterized single-photon emitters. Removal of the unexposed photoresist leads to the final structure. (**e**) Electron micrograph of several fabricated crossed-arc waveguides. Each crossing contains one pre-characterized single-photon emitter. (**f**) Magnified view of one crossing.

Fabrication setup: The home-built setup for localization, selection, and lithography is based on the body of an inverted microscope (DMIRB, Leica). The laser for fluorescence excitation was a linearly polarized continuous-wave (cw) solid-state laser (OBIS 561 LS, Coherent) emitting at 561 nm wavelength. The laser for lithography was a linearly polarized femtosecond laser (T-Light, Menlo Systems GmbH) with center wavelength 780 nm. It was modulated by an acousto-optic modulator (MTS40-A3-750.850, AA Opto Electronic). The two laser beams were focused through the same oil-immersion microscope lens (100×, NA = 1.4, Zeiss). The 100 nm diameter gold beads (BBI Solutions) used for characterization of the foci were embedded in a tri-acrylate photoresist with a thickness of 1.45 mm and clad between two glass cover slides. The quoted excitation powers were measured at the entrance pupil of this focusing lens. For fine sample scanning, we used a 3D piezoelectric stage (PI P-527.3CL, Physik Instrumente) with 200 μm travel in the lateral (*xy*) directions and 20 μm travel in the axial (*z*) direction. For coarse scanning, we used a 2D motorized stage (SCAN IM 120 mm × 100 mm, Märzhäuser Wetzlar). The emission from the sample was collected by the same microscope objective lens and imaged onto a multi-mode optical fiber. For the confocal detection, we used a 15 μm (about one Airy unit) diameter pinhole in an intermedia image plane and a focal length of 5 cm. To suppress the pump light, we used a long-pass filter with a cut-on wavelength of 650 nm (FEL0650, Thorlabs GmbH). The *in-situ* autocorrelation measurements were performed by replacing the fiber by a 50%:50% Y-shaped fiber coupler (MM coupler grade A, Laser Components). The two outputs of the fiber were connected to two single-photon counting modules (TAU-SPAD-100, PicoQuant). For the second-order correlation experiments, the two modules were connected to a time-tagging electronics (TimeHarp 260P, PicoQuant). No background subtraction of any sort was performed. Due to the direct fiber coupling to the detectors, fluorescence arising from detection events in the avalanche photodiodes cannot be filtered out. Hence, the emission from one photodiode gets coupled to the other with a time delay of |*τ*| ≤ 20 ns, leading to corresponding artifact peaks in the *g*^(2)^(*τ*) traces. Thus, for clarity, we only show data for |*τ*| ≤ 20 ns in Figs 1c and 4c,e. Data on an extended time-delay scale are shown in Fig. S2. For large time delays, *g*^(2)^(*τ*) decays to 1. For completeness, we occasionally also recorded PL spectra at selected locations (not depicted), again *via* fiber coupling. As expected for room-temperature operation, the PL spectrum of an NV center in a nanodiamond is dominated by phonon sidebands, resulting in a broad emission in the range from 630 nm to 800 nm wavelength.

**Figure 3:**
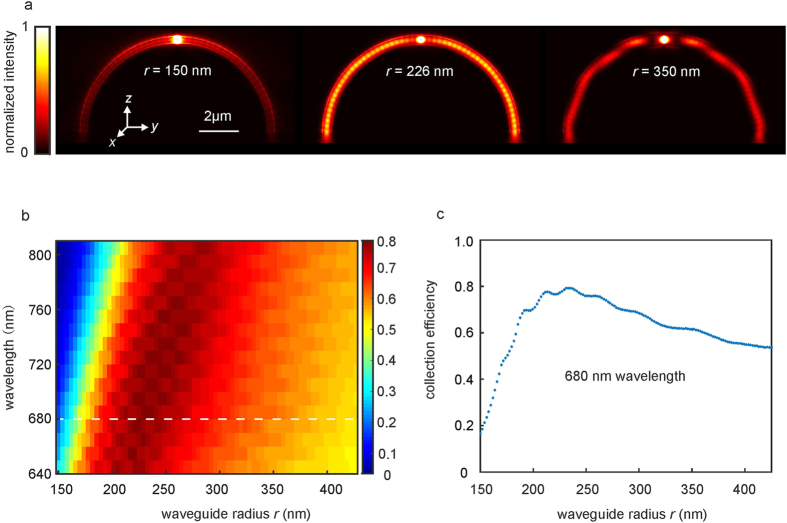
Single-photon collection efficiency. Numerical calculations for crossed-arc waveguide structures with arc radius *R* = 5 μm and waveguide radius *r*. A dipole emitter oriented along the *z*-direction is located in the middle of the crossing. (**a**) Light intensity distribution in a plane parallel to the *yz*-plane cutting through the middle of the waveguide for three different waveguide radii *r* at a vacuum wavelength of 680 nm. (**b**) Collection efficiency versus waveguide radius and wavelength on a false-color scale. (**c**) Detailed scan of collection efficiency versus waveguide radius at a fixed vacuum wavelength of 680 nm.

Numerical calculations: All calculations regarding the arc waveguides used JCMwave, a full 3D frequency-domain finite-element solver. The wavelength-dependent refractive indices of the substrate (polymer) were taken from the Schott glass (PMMA) data sheet. Two tori with radius *R* *=* 5 μm cross under an angle of 180°. The structure is placed on top of a glass substrate and surrounded by air with refractive index *n* = 1. A *z*-oriented dipole (see Fig. 3a) was placed in the middle of the crossing to simulate the emission of an NV center. In order to numerically detect the guided flux through the arc ends, circular regions with a radius of 800 nm were placed on each arc end (*i.e*., where arcs and substrate meet). To calculate the collection efficiency, the flux of light leaving the arc waveguides towards the glass substrate was divided by the total flux leaving the overall surface of the simulation volume *via* the perfectly matched layers (PML).

Measurement setup: We used the same components as in the setup for the fabrication (see above). However, we tilted a mirror in the optical path imaging the sample plane onto the intermediate image plane. In this fashion, we effectively moved the position of the pinhole relative to the intermediate image plane, such that we could pump into any one of the four waveguide ports and collect the emission from any other one of the four ports. For efficient collection via a microscope with an optical axis normal to the substrate plane, the waveguide axes at the substrate needed to be normal to glass substrate plane.

**Figure 4:**
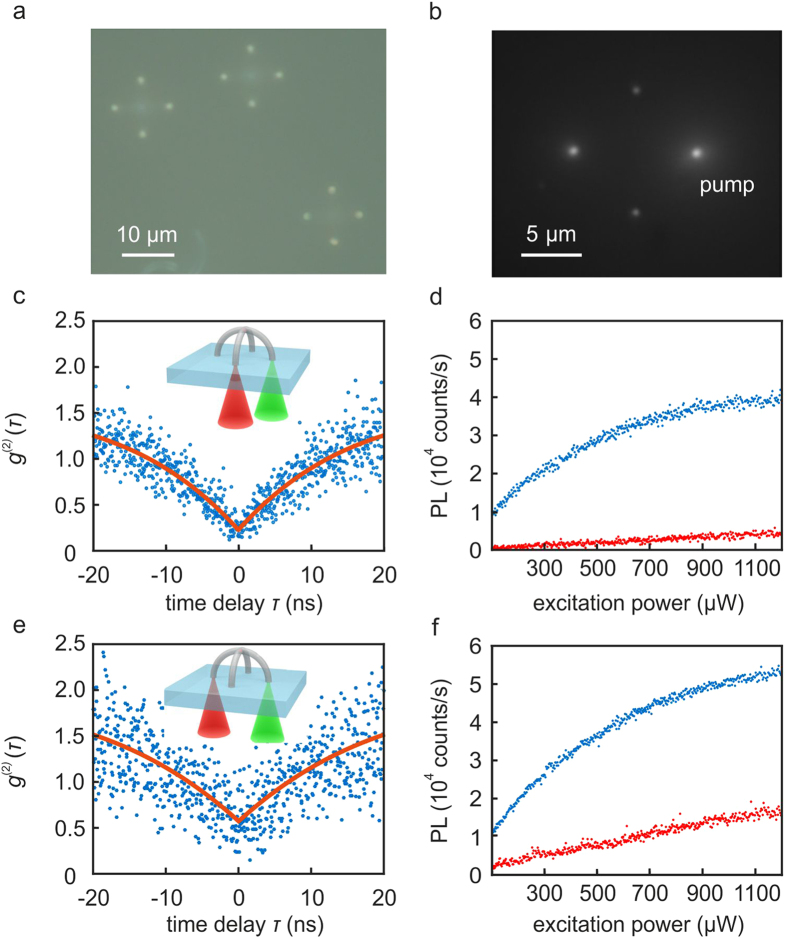
Characterization of single-photon collection from crossed-arc waveguides. (**a**) Wide-field reflection-mode optical microscope image under white-light illumination showing light coupled in and out of the four waveguide ports for three different crossed-arc structures. (**b**) Wide-field fluorescence image of a reference structure *without* a single-photon emitter. Upon pumping into the right port, we observe reduced background photoluminescence (PL) emerging from the orthogonal waveguide. (**c**) Structure *with* a single-photon emitter in the crossing. The blue dots are raw data of 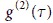 measurements, the red curve is a corresponding fit. Excitation is at 561 nm wavelength and 900 μW incident power. The inset illustrates that detection (red) is from the orthogonal waveguide with respect to the excitation (green). (**d**) The PL saturation curves compare the count rates of the structure with (blue) and a reference structure without (red) a single-photon emitter in the crossing. (**e**,**f**) As panels (**c,d**) but for detection from the excited waveguide, see inset in (**e**).

It was difficult to estimate the in-coupling efficiency of the pump beam into the waveguide. Thus, to obtain the order of magnitude for the collection efficiency of single-photon emission into the double-arc waveguide structure, we compared the measured saturation count rates. These were defined as half of the maximum possible count rates for very large pump power. The saturation count rates were obtained by fitting to saturation measurements like shown in Fig. 4d,f. From Fig. 4d, we obtained a saturation count rate of about 19,000 counts/s for one port, hence roughly 76,000 counts/s for all four ports. For the conditions as in Fig. 1, we obtained typical saturation count rates of 125,000 counts/s for roughly one half of the total solid angle (assuming the emission pattern of a dipole oscillating parallel to the substrate plane), thus 250,000 counts/s roughly estimated for the total solid angle. The collection efficiency was thus about 30%.

## Additional Information

**How to cite this article**: Shi, Q. *et al*. Wiring up pre-characterized single-photon emitters by laser lithography. *Sci. Rep*. **6**, 31135; doi: 10.1038/srep31135 (2016).

## Supplementary Material

Supplementary Information

## Figures and Tables

**Figure 2:**
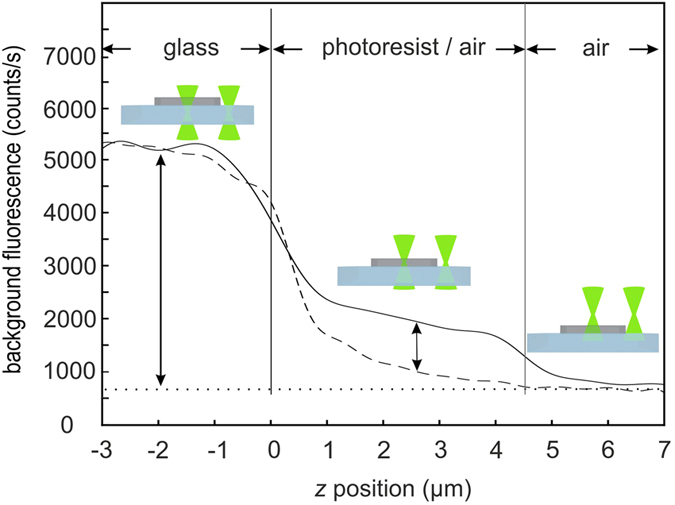
Photoresist with low background fluorescence. A laser written photoresist cube (5 μm side length, without nanodiamonds) on top of a silica glass substrate is excited by a tightly focused (NA = 1.4) continuous-wave laser at 561 nm wavelength and with 80 μW power. The resulting background fluorescence is recorded by confocal detection versus the *z*-position of the sample with respect to the laser focus. The solid curve shows a *z*-scan through the resist, whereas the dashed curve is a *z*-scan through a glass-air interface for comparison. If the focus lies within the glass substrate (*z* < 0 μm), we detect a signal that is much larger than the dark count rate (670 counts/s) for the case that the focus lies in air (*z* > 4.5 μm). The background fluorescence count rate from the photoresist of 1000 counts/s (difference between solid and dashed curves) is about six times smaller than that of the glass substrate.
