# Supplementary material for: Wiring up pre-characterized single-photon emitters by laser lithography
Source: Sci Rep. 2016 Aug 10;6:31135. doi: 10.1038/srep31135 (PMC4979026; doi:10.1038/srep31135)
Supplement: Supplementary Information [file srep31135-s1.pdf]

Wiring up pre-characterized single-photon emitters by laser lithography

Authors: Q. Shi[1], B. Sontheimer[2], N. Nikolay[2], A. W. Schell[3], J. Fischer[4], A. Naber[1], O. Benson[2], and M. Wegener[1,4 *]

Affiliations:

[1]Institute of Applied Physics, Karlsruhe Institute of Technology (KIT), 76128 Karlsruhe, Germany.

[2]Nano-Optics, Institute of Physics, Humboldt-Universität zu Berlin, Newtonstraße 15, D-12489 Berlin, Germany.

[3]Department of Electronic Science and Engineering, Kyoto University, Kyoto Daigaku-Katsura, Nishikyo-ku, 615-8510 Kyoto, Japan.

[4]Institute of Nanotechnology, Karlsruhe Institute of Technology (KIT), 76344 Eggenstein-Leopoldshafen, Germany.

* martin.wegener@kit.edu

Supplementary Information:

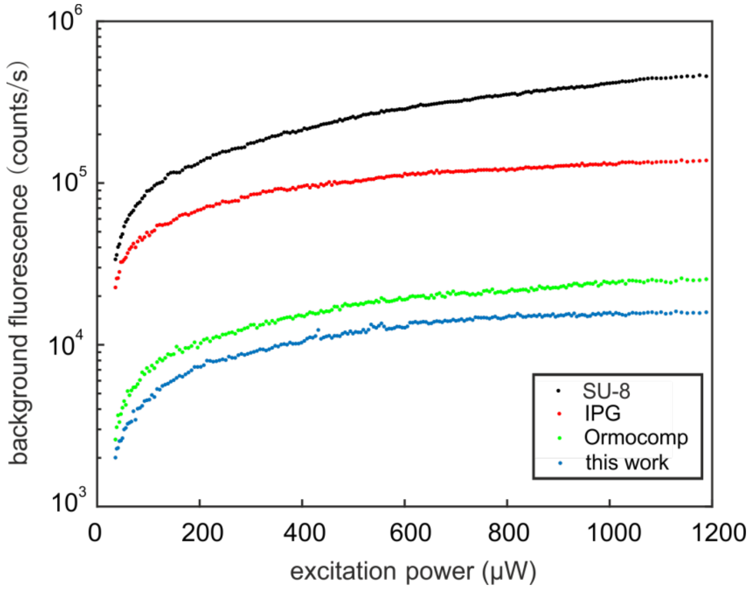

Fig. S1 | Background fluorescence of four different photoresists. SU-8 (black dots) is a commercial photoresist sold by Microchem Corp., IPG (red dots) a commercial photoresist sold by Nanoscribe GmbH, and Ormocomp (green dots) a commercial photoresist sold by micro resist technology GmbH. The blue dots correspond to the home-made solid resist introduced and described in this work. Note that IPG and Ormocomp are liquid prior to polymerization, making them unsuitable for the localization of single-photon emitters in nanodiamonds (see main paper). After drop casting each of the four photoresists on a separate glass substrate (no nanodiamonds), square-shaped areas with 10 μm side length and 6 μm height are exposed and polymerized by our lithography setup. Next, we switch to the excitation laser at 561 nm wavelength and locate its focus within the photoresist volume and record the fluorescence. In each case, we start with high excitation power. We find that the fluorescence starts from a certain level and decreases

versus time (*i.e.*, the emission centers bleach) until the fluorescence intensity reaches a steady state after some minutes. The data shown are within this steady state for each of the four photoresists. The recorded background fluorescence on a logarithmic scale is depicted versus excitation power.

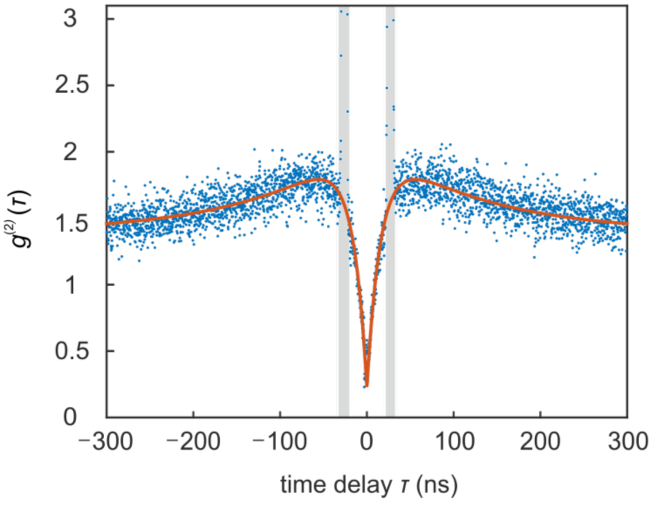

Fig. S2 | Second-order correlation experiment on extended scale. Same measurement as in **Fig. 4c** but shown on an extended time-delay scale (also see Methods). Blue dots are measured data, the red curve is a fit following C. Kurtsiefer *et al.*, *Phys. Rev. Lett.* **85**, 290-293 (2000). The gray areas highlight regions of crosstalk between the two photodiodes. The data in these areas are omitted from the fitting procedure. For large time delays, $g^{(2)}(\tau)$ decays to 1.

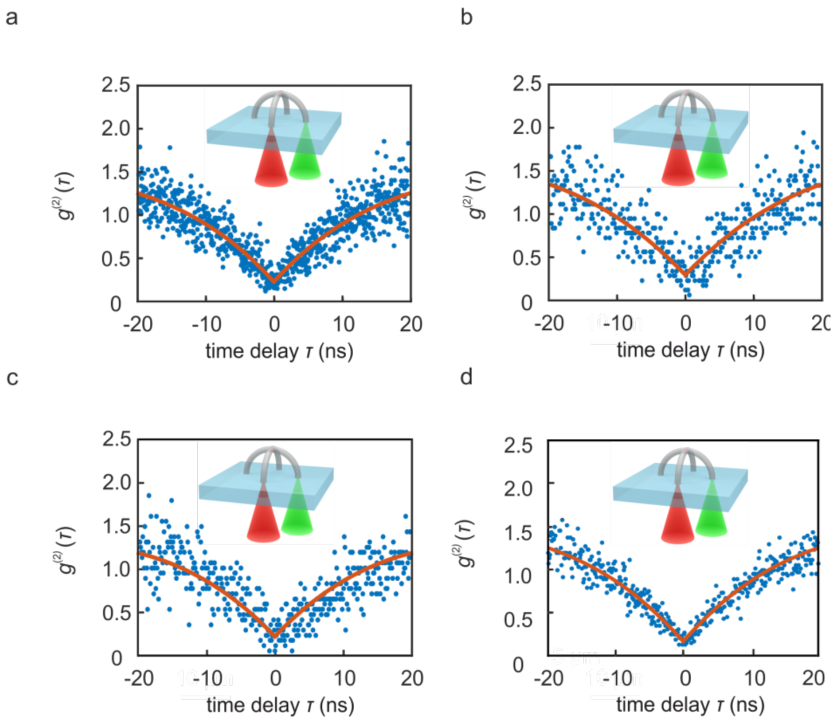

Fig. S3 | Second-order correlation experiment like in Fig. 4c, but for four different samples.

For comparison, panel a is identical to Fig. 4c. These data show that the results shown in Fig. 4 are reproducible.
